# Mechanism of action and potential therapeutic targets of TGF-β-related signaling pathway and its downstream miRNA expression in pulmonary arterial hypertension

**DOI:** 10.3389/fphar.2025.1596767

**Published:** 2025-06-06

**Authors:** Yimo Huang, Wende Ma, Cen Guo, Xiaoling Su

**Affiliations:** ^1^ College of Clinical Medicine, Qinghai University, Xining, Qinghai, China; ^2^ Department of Cardiovascular Medicine, Qinghai Provincial People’s Hospital, Xining, Qinghai, China

**Keywords:** pulmonary hypertension, TGF-β, miRNA, personalized therapy, CRISPR, exosomes

## Abstract

Pulmonary hypertension is a major cardiovascular disease characterized by the persistent elevation of pulmonary artery pressure, leading to vascular remodeling, fibrosis, and endothelial dysfunction. In recent years, the TGF-β signaling pathway and miRNAs have played important roles in the pathogenesis of PH. TGF-β regulates the proliferation, migration and fibrosis of vascular smooth muscle cells through the classical Smad pathway and non-classical pathways such as PI3K/Akt and MAPK. miRNAs such as miR-21, miR-145, and miR-204 play key roles. Among them, miR-21 promotes the proliferation and migration of vascular smooth muscle cells, miR-145 inhibits the overproliferation and fibrosis of vascular smooth muscle cells, and miR-204 alleviates vascular remodeling by inhibiting TGF-β signaling. The combination of CRISPR gene editing and an exosome delivery system can precisely regulate miRNA expression, thus providing new therapeutic targets for pulmonary hypertension.

## 1 Introduction

Pulmonary hypertension (PH) is an major cardiovascular disease, the distinctive hallmark of which is a constant increase in pulmonary artery pressure (Hu et al., 2023). Various pathological changes accompany this disease, such as vascular remodeling, endothelial dysfunction, abnormal proliferation of vascular smooth muscle cells (VSMCs), remodeling of the extracellular matrix (ECM), and fibrosis (Kang K. et al., 2024). VSMCs are located within the vascular wall and are responsible for the contraction and relaxation of blood vessels, thereby regulating vascular tone and blood pressure. The ECM is a complex network structure primarily composed of proteins and glycosaminoglycans, providing support, structural integrity, and facilitating cell signaling (Li et al., 2024). Patient quality of life is significantly affected by these pathological changes, resulting in significant dyspnea, increased fatigue, and decreased exercise tolerance. Furthermore, with a greater burden on the right side of the heart, patients with PH are at risk of right heart failure, which poses a significant threat to their lives (Titz et al., 2024; Agarwal et al., 2024). According to the latest data available for 2024, the annual incidence of PH is approximately 16–20 cases per million people worldwide. This figure reflects the global prevalence of PH, especially in high-risk populations (e.g., the elderly, patients with chronic lung disease, and patients with underlying diseases, such as SLE). The annual incidence of PH varies regionally but shows a yearly upward trend. In addition, the mortality rate of PH continues to rise to approximately 10–15 cases per million people. The mortality rate significantly increases as the disease progresses, especially when PH triggers right heart failure (Liu et al., 2025; Kucherenko et al., 2023). Although currently available treatments, such as pulmonary artery dilators, antiplatelet agents, and diuretics, provide some relief and slow disease progression, there are limitations in improving pulmonary vascular remodeling, fibrosis, and inflammation, and there is no definitive cure.

The pathophysiological mechanisms and pathogenesis of PH are complex. First, early PH often shows signs of endothelial impairment; specifically, once endothelial cells are damaged, the synthesis of vasodilatory factors, such as nitric oxide, is inhibited, while the level of vasoconstrictive factors, such as endothelin, increases significantly, which drives vasoconstriction and increases resistance to blood flow, thus increasing pressure in the pulmonary arteries (Cunningham et al., 2021). Secondly, the proliferation and migration of VSMCs play a crucial role in the development of PH. When signaling pathways such as transforming growth factor-β (TGF-β), phosphatidylinositol 3-kinase/protein kinase B (PI3K/Akt), and mitogen-activated protein kinase (MAPK) are abnormally activated, VSMCs undergo a phenotypic transition from contractile to synthetic, leading to the accumulation of ECM, and collagen and elastin deposition. Over time, the vessel wall thickens and becomes stiffer, and vascular compliance decreases, further increasing pulmonary artery pressure and triggering vascular remodeling (Xiao et al., 2022; Wang et al., 2020). Furthermore, the phenotypic transformation of VSMCs not only promotes cell proliferation but also exacerbates fibrosis, resulting in loss of vascular elasticity and hemodynamic deterioration (Tao et al., 2023). In addition, the interplay between hypoxia, inflammatory response, and oxidative stress promotes vascular fibrosis and stiffness (Yang et al., 2024). The inflammatory response disrupts the vascular endothelial barrier and promotes apoptosis and adverse remodeling of endothelial cells through activation of immune cells and secretion of cytokines, while enhancing the activation of the TGF-β signaling pathway, thereby accelerating the proliferation and migration of VSMCs (Zhu H. et al., 2022). Oxidative stress-activated ROS can directly or indirectly regulate the transcription or stability of miRNAs, which in turn affect the expression of target genes. For example, miR-21 expression is significantly upregulated by oxidative stress. miR-21 enhanced the activation of the TGF-β signaling pathway by targeting TGF-β receptor and Smad7, thereby promoting the proliferation, migration, and phenotypic transformation of VSMCs, and driving vascular remodeling and fibrosis (Benedusi et al., 2024; Gang et al., 2023; Song et al., 2024).

Additionally, PH is influenced by both genetic and epigenetic factors (Adu-Amankwaah et al., 2025). Genetic factors influence the function of the TGF-β signaling pathway by altering the expression of key genes in the pathway. For example, certain single nucleotide polymorphisms (SNPs) in the TGF-β1 gene may lead to upregulation of its expression or alteration of its function, thereby enhancing the activation of TGF-β signaling; this promotes proliferation and migration of VSMCs and drives the progression of PH (Poppelaars et al., 2021). In addition to genetic factors, the role of epigenetic factors (e.g., DNA methylation, histone modification, and non-coding RNA expression) in the TGF-β signaling pathway and miRNA regulation has received increasing attention. For example, the DNA methylation status of TGF-β-related genes such as TGFBR2 and Smad2/3 may affect their expression and thus TGF-β signaling. Studies have shown that hypermethylation or demethylation of certain TGF-β signaling pathway-related genes in patients with PH may lead to abnormal expression of these genes, resulting in different phenotypes in different patients (Ding et al., 2024). Additionally, miRNA expression patterns are regulated by epigenetic factors. For example, the expression of miRNAs such as miR-21 and miR-29 is regulated by DNA methylation, histone modifications, and long noncoding RNAs. In PH patients, upregulation of miR-21 may promote the proliferation and migration of VSMCs by inhibiting negative regulators of the TGF-β signaling pathway, such as Spry1, while downregulation of miR-29 is closely related to the fibrotic process of blood vessels (Zaiou et al., 2021; Han et al., 2023; Almanzar et al., 2023). Therefore, the regulatory mechanisms of miRNAs are being viewed as new potential therapeutic targets for personalized treatment of PH.

## 2 Role of the TGF-β signaling pathway in PH

### 2.1 Mechanisms of the TGF-β signaling pathway and its role in PH

**FIGURE 1:**
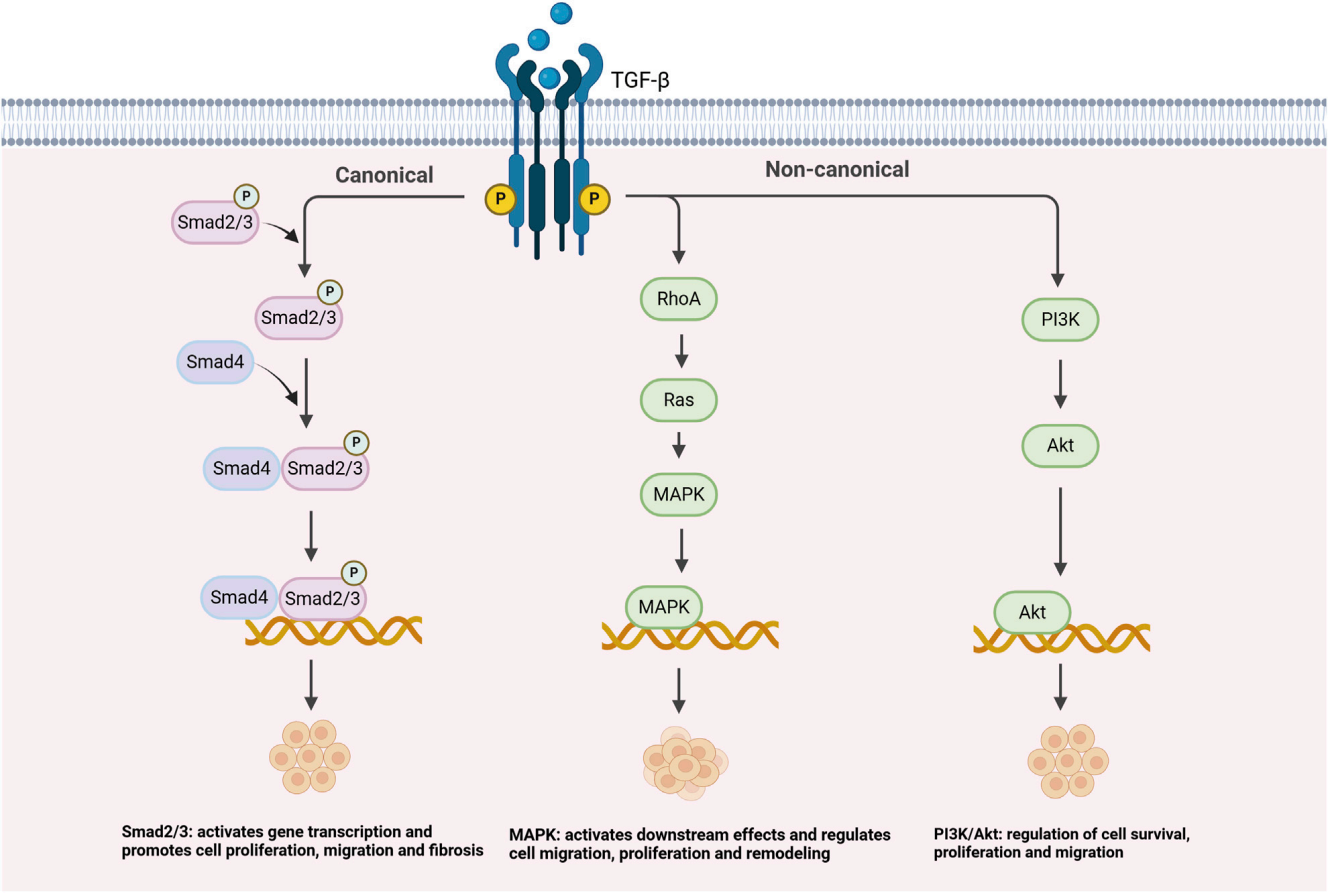
Mechanism of TGF-β signaling pathway in pulmonary arterial hypertension.

As shown in Figure 1, the TGF-β signaling pathway plays a crucial role in the pathogenesis of PH. The regulation of TGF-β signaling is mainly achieved through the classical Smad2/3 pathway, but also through several non-classical pathways, such as PI3K/Akt, MAPK, and RhoA/ROCK, which regulate the proliferation, migration, and fibrosis process of VSMCs, and further contribute to vascular remodeling and PH progression (Shi et al., 2022). Recent studies have shed new light on the complex role played by TGF-β signaling during vascular remodeling and fibrosis, especially in the context of PH. For example, Shi et al. (2022) demonstrated in animal studies that the TGF-β signaling pathway promotes the phenotypic transition of VSMCs in PH by activating the Smad2/3 pathway, thereby exacerbating vascular remodeling and fibrosis. Although this study provides important insights into the mechanisms of PH, it is limited to animal models, and whether similar effects can be observed in clinical studies requires further validation. Additionally, Zhao et al. (2023) observed in *in vitro* experiments how TGF-β signaling interacts with the PI3K/Akt and MAPK pathways to promote VSMC proliferation and migration, thereby accelerating the progression of PH (Liu et al., 2024; Shi et al., 2022; Zhao et al., 2023). Although *in vitro* experiments provide molecular-level insights, translating these results into clinical treatment strategies requires further validation through clinical trials to assess their practical applicability. Although these results may seem different, they actually reveal multiple roles of TGF-β in PH. TGF-β not only affects VSMCs through the classical Smad2/3 signaling pathway, but also promotes vascular remodeling by interacting with non-classical pathways, such as PI3K/Akt and MAPK. Specifically, in the classical Smad2/3 pathway, TGF-β activates the kinase activity of its receptor TGFBR1 by binding to and TGFBR2, which subsequently leads to phosphorylation of Smad2 and Smad3. Phosphorylated Smad proteins then form a complex and move to the nucleus, where they interact with other transcription factors to regulate the expression of a series of downstream genes, particularly those related to fibrosis, such as *COL1A1* and *FN1*. This process promotes the accumulation of ECM, resulting in fibrosis and stiffening of the vessel wall ultimately causing pulmonary artery pressure to rise (Sun et al., 2022). In addition to the classical Smad pathway, TGF-β is also regulated by several other important signaling pathways, including the PI3K/Akt pathway, the MAPK pathway, and the RhoA/ROCK pathway. Specifically, the PI3K/Akt pathway, by inhibiting the expression of apoptotic genes (e.g., *Bcl-2*), resulted in a significant increase in the survival rate of VSMCs and a significant enhancement in proliferation in PH (Yao et al., 2021). By virtue of its regulation of cell proliferation, migration, and differentiation, the MAPK pathway allows greater advancement in vascular remodeling (Mitsis et al., 2024). The RhoA/ROCK pathway, which regulates cytoskeletal reorganization, significantly enhances the migratory capacity of VSMCs and accelerates the process of vascular remodeling and fibrosis, allowing greater exacerbation of clinical symptoms in PH (Zhang et al., 2024; Chouw et al., 2022). The interaction of these classical and non-classical signaling pathways together promotes the pathological progression of PH, allowing VSMCs to shift from a contractile to a synthetic phenotype, worsening vascular fibrosis and stiffness (Xiao et al., 2022; Wang et al., 2020).

### 2.2 Interaction of TGF-β with other signaling pathways and its role in PH

TGF-β does not exert influence on pathological progression in an independent manner, but interacts closely with multiple signaling pathways, resulting in a complex regulatory network that further drives the course of PH. Specifically, the interaction of the TGF-β signaling pathway with the bone morphogenetic protein (BMP) signaling pathway is seen as a key factor in the development of PH (Cullivan et al., 2022). Under normal physiological conditions, it is BMP signaling that effectively inhibits the overproliferation and fibrosis of VSMCs through the activation of BMP receptor type II (BMPR2), thereby maintaining normal vascular function (Correale et al., 2024b). However, when the BMPR2 gene is mutated or absent, impaired BMP signaling results in enhanced TGF-β signaling, which triggers vascular fibrosis and stiffness, and significantly accelerates the progression of PH, especially in patients of PH with BMPR2 mutations (Cullivan et al., 2022). This finding is primarily based on animal model studies, which, although providing strong support for the mechanisms of PH, require further validation through clinical cohort studies to confirm their applicability in clinical settings. In hypoxia, it is the activation of HIF-1α that significantly contributes to the pathogenic mechanism of the TGF-β signaling pathway and promotes vascular stiffness and fibrosis (Li L. et al., 2022). HIF-1α is a transcription factor that activates the expression of multiple genes under hypoxic conditions, regulating cellular adaptation to the hypoxic environment (Gong et al., 2024). HIF-1α is activated and translocated into the nucleus, initiating the transcription of a series of genes, including genes related to angiogenesis, cellular proliferation, metabolic regulation, and fibrosis (Kao et al., 2023). Signaling pathway by directly regulating key molecules, such as Smad2/3, which promotes the proliferation and migration of VSMCs and plays a driving role in the pathogenesis of PH (Zhu N. et al., 2022). In addition, HIF-1α upregulates the expression of miRNAs associated with fibrosis, such as miR-21 and miR-221, which promotes the transformation of VSMCs to a synthetic phenotype and further exacerbates the fibrotic response of the vessel wall (Korvenlaita et al., 2023). Most of these studies are based on *in vitro* experiments and animal models, but their validation in clinical trials requires further investigation. Only after clinical validation can their effectiveness in actual treatment be confirmed. Recent studies have further clarified the crucial role of HIF-1α in PH. For example, Wan et al. (2024) demonstrated in a clinical observational study that HIF-1α plays a significant pathogenic role by regulating the expression of genes associated with pulmonary vascular remodeling. In addition, Maimaitiaili et al. (2023) showed that it is also HIF-1α that exacerbates PH occurrence through the IKK/NF-κB signaling pathway (Wan et al., 2024; Sun et al., 2024; Maimaitiaili et al., 2023). These studies provide new perspectives on the role of HIF-1α in PH, but further clinical trials and cohort studies are needed to confirm its efficacy as a therapeutic target.

## 3 The role of miRNAs in PH

### 3.1 Basic functions and mechanisms of miRNAs

There is a class of non-coding RNAs that are approximately 20–24 nucleotides in length, called miRNAs. miRNAs play an important role in the post-transcriptional regulation of gene expression and can regulate a wide range of biological processes inside and outside the cell (Ajila et al., 2023). Recently, the functions of miRNAs in PH have been investigated in detail. miRNAs are often used to degrade or inhibit the translation of target genes through complementary binding to the 3′ untranslated region (UTR) of target mRNAs, thus realizing the regulation of gene expression (Chien et al., 2021). For example, according to Kipfmueller et al. (2023), miRNAs play an important role in PH associated with congenital diaphragmatic hernia, which provides a potential target for the treatment of this disease. In addition, Kim et al. (2023) highlighted the key regulatory role of non-coding RNAs in cardiac development and cardiovascular diseases, which is likely to be closely related to the development of PH, and Mao et al. (2023) investigated the signaling pathway of the long chain non-coding RNA MALAT1 in the development of lung diseases, and revealed its potential contribution to the development of PH (Pugnaloni et al., 2023; Kawaguchi et al., 2023; Liu J. et al., 2023; Kipfmueller et al., 2023; Kim et al., 2023). The production of miRNAs begins with the transcription process, which is carried out in the nucleus of the cell. During this process, the Drosha enzyme is responsible for shearing, which results in the formation of precursor miRNAs (pre-miRNAs) that are then transported to the cytoplasm by Exportin-5 (Lobera et al., 2023). After reaching the cytoplasm, Dicer enzyme further processes the precursor miRNA into a biologically active double-stranded miRNA (Xu et al., 2024b). After maturation, miRNAs bind to the RNA-induced silencing complex (RISC), which directs the RISC to specifically bind to specific sequences of the target mRNA, thereby regulating gene expression by inhibiting translation or promoting mRNA degradation (Meng et al., 2021). RISC is an intracellular protein-RNA complex that regulates gene expression by binding to miRNA, inhibiting the translation of target mRNA or promoting its degradation (Musumeci et al., 2022). Abnormalities in RISC expression are associated with the development of various diseases such as cancer, cardiovascular disease, and PH (Zhao et al., 2023).

**FIGURE 2:**
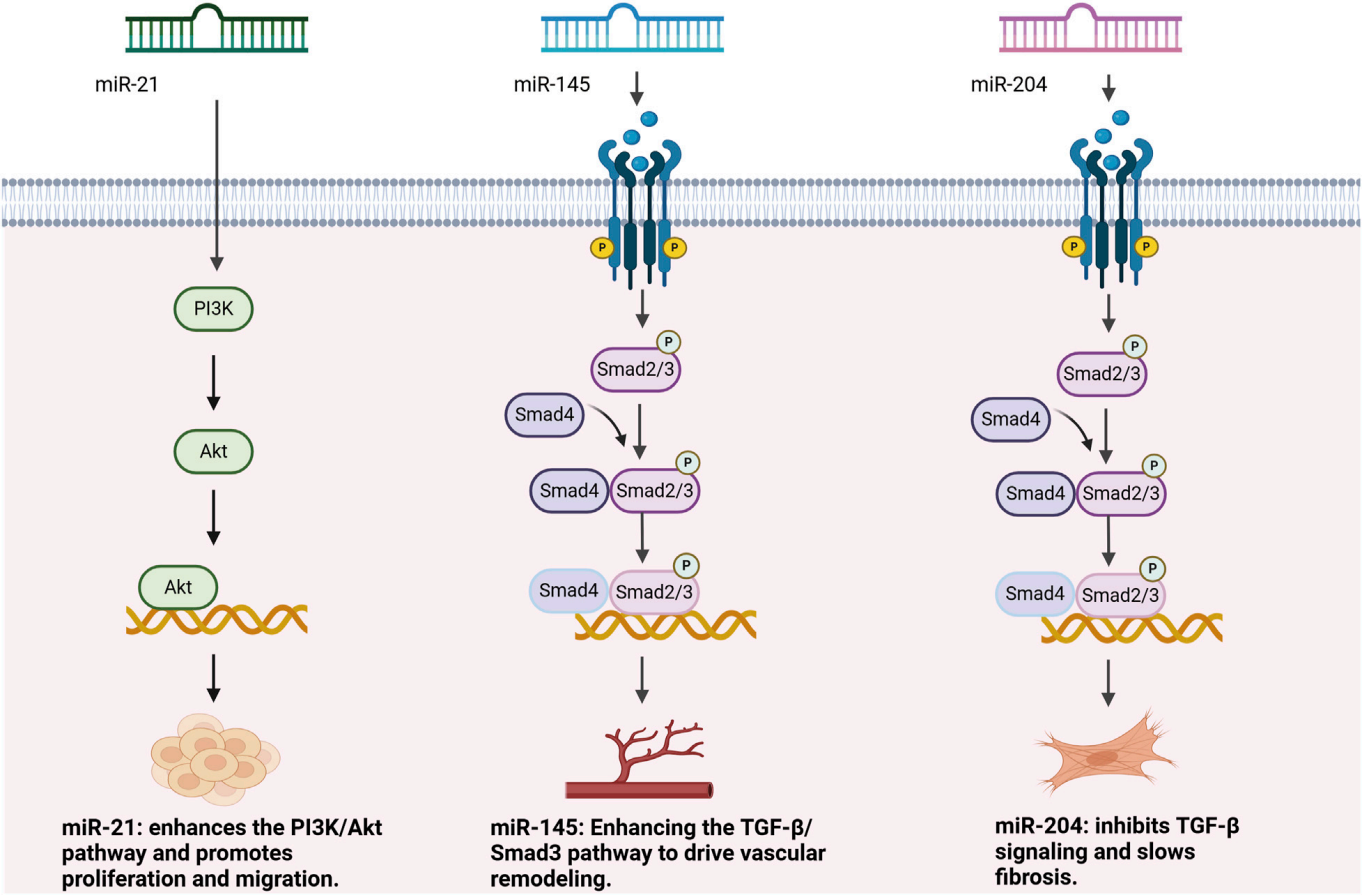
The role of miRNAs in pulmonary arterial hypertension.

In recent years, miRNAs have been identified as key molecules in the regulation of TGF-β signaling, especially through upstream and downstream regulation to fine-tune this signaling pathway and promote or inhibit vascular remodeling and epithelial mesenchymal transition (EMT) (Kang J. et al., 2024). EMT is a cellular biological process in which epithelial cells lose their polarity and adhesiveness, transitioning into mesenchymal cells with migratory and invasive properties, playing a crucial role in processes such as tumor progression and fibrosis (Shin et al., 2023). Based on available miRNA microarray data, multiple miRNAs have been shown to play important roles in the TGF-β signaling pathway (El Hayek et al., 2024). As shown in Figure 2 and Table 1, upstream of the TGF-β signaling pathway, miRNAs have an important impact on signaling by regulating the TGF-β receptor and its precursor molecules. For example, miR-21, as an upstream miRNA, directly or indirectly enhances signaling by targeting negative regulators in the TGF-β signaling pathway. For example, miR-21 enhances the activation of the TGF-β signaling pathway by regulating the PI3K/Akt pathway, thereby promoting responses such as cell proliferation, migration, and fibrosis (Eid et al., 2021). Downstream of the TGF-β signaling pathway, multiple miRNAs exert their regulatory effects by directly regulating Smads and other key effector molecules. For example, miR-204 and miR-145 are considered downstream regulators of the TGF-β signaling pathway. miR-204 alleviates vascular remodeling by inhibiting the activity of TGF-β receptors and Smad2, thereby slowing the proliferation and fibrosis of vascular smooth muscle cells. miR-145 promotes the proliferation, migration, and vascular remodeling of vascular smooth muscle cells by upregulating the expression and phosphorylation of Smad3, enhancing the activation of the TGF-β/Smad3 signaling pathway (Tang et al., 2022; Han et al., 2024).

**TABLE 1:** Role and clinical application of TGF-β/miRNA in PH.

Signaling pathway/miRNA	Mechanism of action	Role in PH	Latest therapeutic targets and clinical applications	References
TGF-β signaling pathway	Activation of classical Smad2/3 pathway and non-classical PI3K/Akt and MAPK pathways promotes VSMC proliferation, migration, and fibrosis.	TGF-β causes right heart failure by enhancing the proliferation, migration, and fibrosis of VSMCs, leading to thickening and stiffening of the vessel wall and elevating pulmonary artery pressure.	Targeted therapy: targeting TGF-β receptor or its downstream signaling pathways (e.g., Smad2/3, PI3K/Akt) to slow down vascular remodeling through anti-fibrosis and anti-inflammation. Repair TGF-β receptor mutations or downstream signaling pathway abnormalities using CRISPR technology.	Shi et al. (2022)
miR-21	Activation of PI3K/Akt signaling pathway enhances VSMC proliferation, migration and inhibits apoptosis.	Upregulation of miR-21 promotes cell proliferation and migration, exacerbates fibrosis, vascular sclerosis, and drives the progression of PH.	Targeted therapy: Inhibit its overexpression through anti-miR-21 therapy to slow down vascular fibrosis, proliferation and hardening. Combined with the exosome delivery system, the precise delivery of inhibitors significantly improves the therapeutic effect.	Eid et al. (2021)
miR-145	Enhancement of the TGF-β/Smad3 pathway drives vascular fibrosis and remodeling and regulates ECM accumulation.	miR-145 enhances vascular sclerosis in PH by promoting vascular remodeling, leading to elevated pulmonary artery pressure.	Targeted therapy: restoration of miR-145 expression to inhibit the process of vascular remodeling and fibrosis. Targeting miR-145 binding to TGF-β/Smad3 pathway to slow down vascular stiffness. Adjusting miR-145 levels using gene editing technology.	Han et al. (2024)
miR-204	Inhibition of TGF-β signaling pathway overactivation reduces fibrosis and stiffness of blood vessels.	Downregulation of miR-204 exacerbates TGF-β signaling activity, leading to increased vascular fibrosis and stiffness with PH.	Therapeutic target: restoring miR-204 expression, slowing down the process of vascular fibrosis and sclerosis, and delaying PH progression. Exosome delivery system is used to precisely repair miRNA function defects and enhance therapeutic effects.	Tang et al. (2022)

### 3.2 miR-204: a protective factor in PH

miR-204 plays a crucial protective role in the development of PH, especially in processes involving vascular remodeling and fibrosis (Upreti et al., 2024). Studies have shown that miR-204 can play an effective role in slowing the proliferation, migration, and anti-apoptotic properties of VSMCs by targeting the Src and STAT3 signaling pathways, which have a significant and positive impact on curbing the process of vascular remodeling and fibrosis (Tang et al., 2022). miR-204 directly targets fibrosis-related genes such as *COL1A1* and *FN1*, which are involved in the accumulation and deposition of ECM. By inhibiting their expression, miR-204 reduces ECM deposition, thereby maintaining vascular elasticity and normal function. Under normal physiological conditions, miR-204 effectively regulates the expression of these genes, suppressing fibrosis and preserving the elasticity of the vascular wall (Dong et al., 2024). However, in patients with PH, miR-204 expression levels are significantly reduced, resulting in a weakened inhibitory effect on antiapoptotic genes (e.g., *Bcl-2*), which contributes to the aberrant proliferation and phenotypic transformation of VSMCs, accelerates vascular wall remodeling and fibrosis, and advances the progression of PH (Zehrfeld et al., 2024). Loss of miR-204 function not only interferes with the normal regulatory process of the ECM but also aggravates vascular stiffness and stiffens the vasculature^.^ However, it exacerbates the degree of vascular stiffness and decreases elasticity, further aggravating the associated clinical symptoms. In PH, by down-regulating the expression of miR-204, HIF-1α caused a significant increase in the progression of PH. Therefore, normalization of miR-204 expression is appropriate as a potential therapeutic strategy for PH. Studies have shown that restoration of miR-204 is effective in reducing the proliferation of VSMCs, inhibiting cell migration, and facilitating the reconstruction of normal vascular structures, thereby alleviating the clinical symptoms of PH (Correale et al., 2024a). Notably, the function of miR-204 is not limited to pulmonary artery smooth muscle cells (PASMCs) but also plays an important role in the adaptive remodeling of the right heart. Studies have shown that downregulation of miR-204 in the right heart tissues of patients with PH exacerbates cardiomyocyte apoptosis and fibrosis, driving the progression of right heart hypertrophy. miR-204 inhibited cardiomyocyte proliferation and phenotypic transformation by targeting TGF-β receptor and Smad2, thereby slowing down the right heart hypertrophy and preserving right heart function (Upreti et al., 2024; Kinget et al., 2021). In addition, overexpression of miR-204 in an animal model significantly improved the pathology of PH and reduced the elevated pulmonary artery pressure and increased burden on the right heart, which is valuable for providing new research directions for the targeted therapy of PH (Karabaeva et al., 2024). However, although overexpression of miR-204 has shown significant improvements in animal models, more preclinical studies and clinical trials are needed to assess the potential side effects associated with miR-204 overexpression. To ensure the therapeutic efficacy and safety of miR-204, future research should focus on more precise targeted delivery systems, such as nanoparticle or viral vectors, to regulate its expression in specific cell types and avoid unintended effects on other cellular functions.

### 3.3 miR-145: a driver of vascular remodeling

Studies have shown that under normal physiological conditions, miR-145 inhibits VSMC phenotypic transition from a synthetic to a migratory phenotype by downregulating KLF4 expression, thereby suppressing vascular remodeling and fibrosis. However, in PH patients, miR-145 expression is significantly increased. Specifically, by enhancing the activation of the TGF-β/Smad signaling pathway, miR-145 promotes the translocation of Smad3 to the nucleus, thereby activating the expression of fibrosis-related genes. This mechanism accelerates the process of vascular fibrosis and stiffening, leading to thickening and hardening of the vessel wall, significantly reducing vascular elasticity and compliance, which subsequently results in elevated pulmonary arterial pressure and increased right heart strain (Han et al., 2024). Studies have shown that the upregulation of miR-145 contributes to adaptive remodeling of the right heart, improves right heart hypertrophy, and promotes of right heart function (Sala et al., 2020; Farani et al., 2022). In addition, miR-145 acts in concert with hypoxia-inducible factor 1α (HIF-1α), which not only enhances the robust expression of miR-145, but also smooths the regulation of multiple downstream targets. The pathological effects of vascular remodeling are exacerbated, leading to the worsening of fibrosis and stiffness, which drives the development of PH to a certain extent (Zang et al., 2021). Relevant studies have shown that the overexpression of miR-145 is closely associated with elevated pulmonary artery pressure and right heart insufficiency in patients with PH, further confirming that miR-145 plays an important role in the pathogenesis of PH (Sala et al., 2020). It is clear that inhibition of miR-145 has become an important direction for PH therapeutic research. Relevant studies have shown that miR-145 inhibitors significantly slow down the rise in pulmonary artery pressure, improve right heart function, reduce vascular remodeling and fibrosis effectively, and help restore vascular compliance (Deshpande et al., 2022). However, the safety assessment of long-term use of miR-145 inhibitors remains insufficient, and miRNA interventions may induce unknown side effects. To ensure the safety of miR-145 inhibitors, future research should include long-term toxicity studies in preclinical animal models to assess the effects of miR-145 inhibitors on different organs and systems, particularly the potential side effects on the immune and cardiovascular systems. Additionally, precise delivery systems and cell-type-specific regulation strategies may help reduce systemic side effects, enabling the safe and effective clinical application of miR-145 inhibitors.

### 3.4 miR-21: an amplifier of inflammation and fibrosis

Relevant studies have shown that miR-21 expression is significantly elevated in VSMCs and other related cells in patients with PH. This overexpression is closely related to the enhancement of cell proliferation, migration, and migration ability as well as anti-apoptotic ability, and also to the intensification of the vascular remodeling process (Mishra et al., 2022). Specifically, miR-21 targets PTEN to suppress its expression, relieving its inhibition of the PI3K/Akt pathway, thereby activating the PI3K/Akt signaling pathway and promoting VSMC proliferation and migration (Eid et al., 2021). Meanwhile, miR-21 enhanced the TGF-β/Smad2/3 pathway activity more significantly, which further amplified the proliferation, migration, fibrosis and inflammatory response of VSMCs (Song et al., 2024). Additionally, miR-21 plays a significant role in regulating right-sided heart function. miR-21 can also contribute to the release of inflammatory factors, such as IL-6 and TNF-α, which leads to an increased local inflammatory response, promotes fibrosis and hypertrophy in the right heart, and exacerbates the progression of PH (Wang J. et al., 2024; Shahraki et al., 2023). In addition, in endothelial cells, miR-21 exacerbates structural changes in the vessel wall by promoting the proliferation and migration of vascular endothelium. However, overexpression of miR-21 may lead to excessive fibrotic response, increase vascular permeability and aggravate symptoms of PH (Xie et al., 2022; Aseervatham, 2023). In a hypoxic environment, activation of HIF-1α significantly increases miR-21 expression levels, a phenomenon that further reinforces the pathogenic effects of TGF-β signaling, thereby accelerating cell proliferation and anti-apoptotic processes, and exacerbating vascular fibrosis, ultimately contributing to the development of PH (Korvenlaita et al., 2023). Therefore, a therapeutic regimen targeting miR-21 is a PH treatment strategy. In preclinical animal models, anti-miR-21 therapies have been shown to significantly reduce the levels of intravascular inflammatory factors and fibrosis markers (e.g., *COL1A1*) by inhibiting the high expression of miR-21, improving pulmonary artery pressure and right heart function, and slowing vascular remodeling (Shao et al., 2022). These findings suggest that it may be feasible to target miR-21 as an innovative approach for treating PH. However, miR-21 is involved in important physiological functions in various cell types, and long-term inhibition may disrupt other physiological processes. In preclinical and clinical studies, it is crucial to carefully assess the potential side effects of prolonged use of miR-21 inhibitors, particularly their impact on the cardiovascular and immune systems. Therefore, developing targeted delivery systems, such as cell-type-specific delivery platforms, to help reduce systemic side effects will be particularly important.

## 4 Interaction between TGF-β and miRNAs and feedback mechanisms

### 4.1 Regulation of miRNAs by TGF-β

In a related study, it was found that TGF-β can drive the developmental process of PH through regulation of miRNA expression (Shi et al., 2022). Specifically, TGF-β achieves upregulation of miR-21 to a great extent. In this process, the expression of fibrosis-related genes (e.g., *COL1A1*) as well as inflammatory factors (e.g., IL-6 and TNF-α) is significantly induced, resulting in a significant increase in the proliferative and anti-apoptotic capacity of PASMCs. Consequently, fibrosis and sclerosis of the vascular wall are aggravated, accelerating the deterioration of PH (Wang and Zheng, 2021). In addition, TGF-β plays a critical role in regulating miR-204 expression. Under normal physiological conditions, miR-204 regulates TGF-β signaling through a negative feedback mechanism (Upreti et al., 2024). However, in the context of PH, the expression level of miR-204 decreases, which results in deregulation of the inhibitory effect on TGF-β signaling, which further aggravates fibrosis and stiffness of the vessel wall (Zehrfeld et al., 2024). Therefore, TGF-β, by virtue of its regulation of miRNA expression, significantly enhances pathogenicity and exacerbates vascular fibrosis as well as inflammatory responses, contributing to the continued progression of PH. These findings provide a theoretical foundation for miRNA therapeutic strategies, suggesting that restoration of normal expression of miR-204 and miR-21 may be a potential intervention for the treatment of PH, and thus open up new research directions for targeting miRNAs for the treatment of PH.

### 4.2 Feedback effects of miRNA on TGF-β signaling

In studies related to the TGF-β signaling pathway, there is a feedback role played by miRNAs. Specifically, these miRNAs regulate the expression and activity of TGF-β signaling, forming a complex feedback loop that further contributes to the pathology of PH. Taking miR-21 as an example, it activates the PI3K/Akt signaling pathway through the inhibition of the tumor suppressor PTEN, resulting in significant enhancement of the proliferation, migration, and anti-apoptotic ability of VSMCs (Eid et al., 2021). Studies have shown that miR-21 expression is significantly upregulated in VSMCs from patients with PH and promotes cell survival and proliferation by activating the PI3K/Akt pathway, further accelerating vascular remodeling and fibrosis (Mishra et al., 2022). At the same time, miR-204 is able to attenuate the pathological effects of TGF-β signaling by effectively inhibiting its activity (Kinget et al., 2021). However, in PH, the expression level of miR-204 was downregulated, which led to weakening of its inhibitory effect on TGF-β signaling. This amplified the activation effect of TGF-β and further promoted the abnormal proliferation of VSMCs and vascular remodeling, accelerated the process of vascular fibrosis and stiffness, and further contributed (Zehrfeld et al., 2024). In addition, during EMT, the miR-200 family inhibits the progression of PH by reversing the process of epithelial-to-mesenchymal cell transformation through the regulation of ZEB1/2 and E-cadherin (Ahmadi-Hadad et al., 2024). Thus, these feedback mechanisms provide a new research perspective and theoretical support for targeted miRNA therapy. In particular, restoring the normal expression of miR-204 or inhibiting the overexpression of miR-21 will most likely become a proven strategy for the treatment of PH.

## 5 The future of personalized treatment in PH

### 5.1 The need for personalized treatment in PH

In the field of PH, personalized treatment is becoming increasingly important, particularly in the context of rapid development of precision medicine. The core idea is to tailor the treatment to the patient’s genetic information, environmental factors, and pathological characteristics to improve its effectiveness (Zhao and Jiang, 2024). Although all patients with PH share common features of elevated pulmonary artery pressure and vascular fibrosis, there are significant individual differences in the pathological characteristics and response to treatment (Wang and Wang, 2024a). Patients vary greatly in many aspects such as miRNA expression levels, regulation of TGF-β signaling pathway, immune response mechanisms, and genetic background, which have a direct impact on disease onset, progression, and response to therapy. With the continuous development and advancement of genomics, transcriptomics, and other multi-omics technologies, researchers are now able to analyze molecular mechanisms in different patients. Key genes, miRNAs, and signaling pathways associated with PH can be clearly identified, and personalized treatment strategies can be developed for these patients (Wang et al., 2023). Precision medicine has the advantage of being able to accurately tailor therapeutic activities to a patient’s specific biomarkers, which effectively avoids one-size-fits-all treatments, reduces the side effects of treatments to a desirable degree, and improves the effectiveness of treatments (Baptiste et al., 2021). The limitations of universal treatment can be effectively countered through precise intervention in the expression of the TGF-β signaling pathway and its downstream miRNAs as well as by providing patients with personalized treatment plans (Zhuang et al., 2021). MicroRNAs in PH, from Pathogenesis to Diagnosis and Treatment provides a comprehensive and in-depth review of the functions of miRNAs in PH and analyzes their potential for disease pathogenesis, diagnosis and treatment. It also analyzes in detail the potential of miRNAs in disease pathogenesis, diagnosis and treatment, providing important theoretical support for personalized therapeutic strategies (Xu et al., 2022).

### 5.2 Potential and challenges of miRNA-targeted therapy

The potential of PH for the treatment of PH is remarkable. Taking the restoration of miR-204 expression and the inhibition of miR-21 overexpression as examples, the results of this study are particularly interesting (Pawluczyk et al., 2021; Shao et al., 2022). Studies have shown that restoration of miR-204 function during the early stages of PH significantly slows disease progression. Targeting miR-21 to inhibit the proliferation and migration of VSMCs can reduce pulmonary arterial pressure and improve right heart function. Although the therapeutic potential of miRNA-targeted therapies is obvious, there are still many challenges to their clinical application (Tang et al., 2022; Shao et al., 2022; Guo et al., 2022). First, the targeting and stability of miRNA delivery systems must be sufficient to ensure that they can be delivered to target cells accurately and avoid triggering nonspecific side effects (Chen et al., 2024). The current delivery system has a series of problems such as inefficiency, poor targeting, and tendency to induce an immune response, which has severely limited the wide application of miRNA therapy (Wu et al., 2022). Second, miRNAs play a complex role in systemic regulation and may affect the expression of multiple genes and signaling pathways. Therefore, the safety and long-term efficacy of miRNAs must be carefully evaluated at the time of application (Abdallah et al., 2021). However, with the advancement of delivery technologies and deepening of clinical research, the application of miRNA-targeted therapies in PH has shown great promise, especially in terms of improving therapeutic specificity and precision (Choi, 2025). For example, the targeted intervention strategies of miR-21, miR-145 and miR-204 can effectively regulate the activity of the TGF-β signaling pathway, thereby improving the clinical symptoms of PH and slowing down its progression (Correale et al., 2024a; Deshpande et al., 2022; Shao et al., 2022). Choi (2025) investigated the role of miRNAs in angiogenesis and proposed a strategy to apply miRNAs in targeted therapies, which may open up new pathways for the treatment of vascular-related diseases such as PH (Choi, 2025).

### 5.3 Prospects for clinical translation and personalized therapy

In the field of PH therapeutics, miRNA-targeted therapies have undergone a challenging journey, from laboratory research to clinical applications in patients. Although these have demonstrated significant therapeutic potential in the laboratory, there are many technical and clinical obstacles to their clinical application (Guo et al., 2022). Currently, efficient and specific miRNA delivery systems are indispensable to ensure that therapeutic molecules are precisely delivered to target cells, avoiding off-target and nonspecific side effects (Chen et al., 2024). In this context, exosome delivery and gene editing technologies (e.g., CRISPR) have brought new solutions. The low immunogenicity and strong cellular targeting ability of exosomes as miRNA carriers significantly improve their therapeutic effects. The CRISPR technology provides the possibility of long-term intervention that can precisely regulate miRNA expression and target gene function (Gu et al., 2024; Tong et al., 2021). In addition, the key point of personalized treatment is to precisely design treatment based on the patient’s genetic background, pathological characteristics, and response to treatment (Boxhammer et al., 2024). With continued advances in genomics, transcriptomics, single-cell technologies, and high-throughput screening, clinicians are now able to more accurately identify differences in miRNA regulation between patients with different PH and tailor highly personalized therapies for each patient (Wang et al., 2023). This type of personalized treatment based on an individual patient’s condition can help improve the accuracy of treatment, effectively reduce unnecessary side effects, and ensure that patients obtain the most appropriate treatment options with stability (Su et al., 2024). Therefore, future research should focus on further optimizing the delivery system and personalized treatment plan, as well as strengthening the collaboration between preclinical research and clinical trials to promote miRNA-targeted therapy as an important and indispensable modality in PH treatment.

## 6 Prospects for emerging technologies in PH treatment

### 6.1 Applications of CRISPR gene editing technology

In the field of PH treatment, the CRISPR gene editing technology has shown remarkable potential, particularly for the precise repair of miRNA expression defects (Liu B. et al., 2023). With the help of CRISPR, researchers can precisely edit miRNA genes associated with PH and restore or regulate the expression of these miRNAs. Thus, the pathological processes caused by miRNA dysregulation can be reversed or slowed down significantly (Li J. et al., 2022). The repair or inhibition of these miRNAs using the CRISPR technology is highly targeted, and the goal of precisely regulating PH pathogenic signaling pathways has been accomplished (Hussen et al., 2023). For example, the CRISPR technology can be used to restore miR-204 expression. Restoration of miR-204 expression can help slow the fibrosis and sclerosis of blood vessels, thereby improving their structure and function of blood vessels (Pawluczyk et al., 2021). In addition, CRISPR technology also has the ability to inhibit miR-21 overexpression, thereby alleviating the proliferation and migration of VSMCs and reducing vascular remodeling and fibrosis, which provides a new idea for the treatment of PH (Shao et al., 2022). Huang et al. (2024) explored the application of CRISPR-Cas9 gene editing technology in combination with an exosomal delivery system and demonstrated the potential of this method in the treatment of PH demonstrating an innovative presentation of gene editing fusion mRNA therapeutic strategy (Huang et al., 2024). Especially in PH animal models, the use of CRISPR technology to restore the expression of specific miRNAs has shown promising therapeutic potential. For example, restoring the normal expression of miR-145 or miR-21 reduces VSMC proliferation and migration, thereby inhibiting the process of vascular remodeling (Palmieri et al., 2023). However, despite its significant therapeutic effects in precisely repairing miRNA expression defects and modulating pathological signaling pathways, its widespread application still faces some challenges. Off-target effects are important issues in CRISPR technology. Off-target effects refer to the possibility that CRISPR technology, when targeting specific genes, may inadvertently edit non-target genes, leading to unexpected genetic alterations (Zeng et al., 2024). Although existing studies and technologies (e.g., optimization of Cas9 proteins and design of more precise gRNAs) have reduced off-target effects, their long-term safety and efficacy need to be verified in a large number of preclinical and clinical studies. Currently, the production cost of CRISPR technology is high, especially in the areas of gene editing vectors, purification of Cas9 proteins, and synthesis of gRNAs, all of which require substantial financial investments and technical support. These costs limit the application of gene editing therapy in small-scale clinical trials, especially in resource-poor regions that may face greater economic pressure (Liu et al., 2020; Mortoglou et al., 2022). In terms of scalability, the CRISPR technology currently faces challenges, especially in clinical treatments, where key issues remain in ensuring the generalizability of the technology across different disease models and in effectively translating successes in the laboratory into large-scale treatment protocols. Although some gene editing treatments have achieved remarkable results in animal experiments, the feasibility and generalizability of CRISPR treatments need to be further evaluated for clinical dissemination, especially in different populations and disease contexts (Xu et al., 2024a). Second, ethical issues are particularly prominent in the application of gene editing technology. Although the CRISPR technology offers unprecedented opportunities for the treatment of PH, permanent alterations in the human genome may trigger ethical controversies. Gene editing may not only affect an individual’s health but may also create genetic variants that can be passed on to future generations, thereby altering the human gene pool. This makes gene editing, especially of embryos and germ cells, a focus of attention for the scientific community, ethicists, and the public (Wang R. et al., 2024). Additionally, the challenges of gene editing in terms of *in vivo* stability cannot be ignored (Nie et al., 2022). Although the CRISPR technology can realize effective gene editing *in vitro*, delivery efficiency and stability *in vivo* remain key challenges in achieving clinical translation. Studies have shown that the reparative effects of gene editing may diminish over time, indicating that the treatment durability may be limited (Chen L. et al., 2025). To address this issue, researchers are exploring ways to enhance the stability of gene editing effects by improving editing tools or employing self-repair mechanisms. Future research should focus on addressing these issues, optimizing the precision and safety of gene-editing technology, and developing relevant ethical norms to ensure the proper use of gene editing in the treatment of diseases such as PH.

### 6.2 Advantages and applications of exosome delivery systems

Exosomal delivery systems have many advantages and applications for miRNA delivery, especially for the treatment of PH (Jadamba et al., 2024). As an miRNA delivery vehicle, it has obvious advantages in the treatment of PH. Its low immunogenicity has a significant effect on the reduction of immune system rejection, which leads to improved therapeutic safety. This is because exosomes are derived from cell membranes and are naturally biocompatible, effectively avoiding the immune response and toxicity problems often associated with traditional drug delivery systems, thereby improving patient tolerance (Wang et al., 2024d). Exosomes exhibit a high degree of targeting and can deliver miRNAs specifically to target cells (Wang et al., 2024c). In this process, by virtue of the combination of specific ligands or receptors on the surface of the exosome and the target cell receptors, the precise delivery of miRNA in the body can be realized, which not only ensures the high efficiency of the treatment but also reduces side effects a lot (Guo et al., 2023). Studies have shown that exosomes can cross biological barriers such as the blood-brain barrier, delivering miRNAs precisely to pulmonary artery smooth muscle cells in PH patients, thereby exerting therapeutic effects (Guan et al., 2024). Compared with traditional delivery systems, the application of exosomes can significantly improve the therapeutic effect and reduce the impact on non-target cells. For example, engineering exosomes to design surface ligands targeting specific pathological states allows for more precise targeting of specific cells or tissues, providing new possibilities for the personalized development of gene editing therapies (Chen W. et al., 2025). Exosomes have a unique ability to cross biological barriers, allowing them to efficiently deliver miRNAs to cells and tissues that are difficult to target, as is the case with hard-to-reach target cells such as PASMCs (Zou et al., 2023). By fusing with the cell membrane, exosomes directly release their internal contents into the cell to enhance their therapeutic efficacy (Shi et al., 2024). Loading miRNA mimics or interfering molecules into exosomes can precisely regulate disease-causing signaling pathways, thereby slowing down vascular fibrosis and inflammation and improving vascular remodeling (Li et al., 2021). Studies have shown that exosome-mediated delivery of miR-204 effectively inhibits VSMC proliferation and migration, thereby slowing the progression of vascular fibrosis and significantly alleviating the clinical symptoms of PH (Tang et al., 2022; Shao et al., 2022). Zhao et al. (2024) explored the potential of exosomes derived from MSCs as gene delivery vehicles, focusing on their combination with CRISPR/Cas9 technology to further enhance the therapeutic efficacy of PH (Zhao et al., 2024). Although exosomal delivery systems show great promise, they face many challenges in practical applications, such as high production costs, insufficient quality control, and large-scale production (Zanganeh et al., 2023). Simultaneously, challenges in *in vivo* delivery efficiency and drug release control must be resolved (Chen et al., 2022). With the advancement of nanotechnology, engineering of exosomes, and continuous improvement of the delivery system, exosomes as miRNA delivery carriers will have broader application prospects in PH therapy in the future and are expected to become an important tool for personalized therapy and precision medicine to provide more accurate treatment strategies.

### 6.3 Prospects for CRISPR binding to exosomes

**FIGURE 3:**
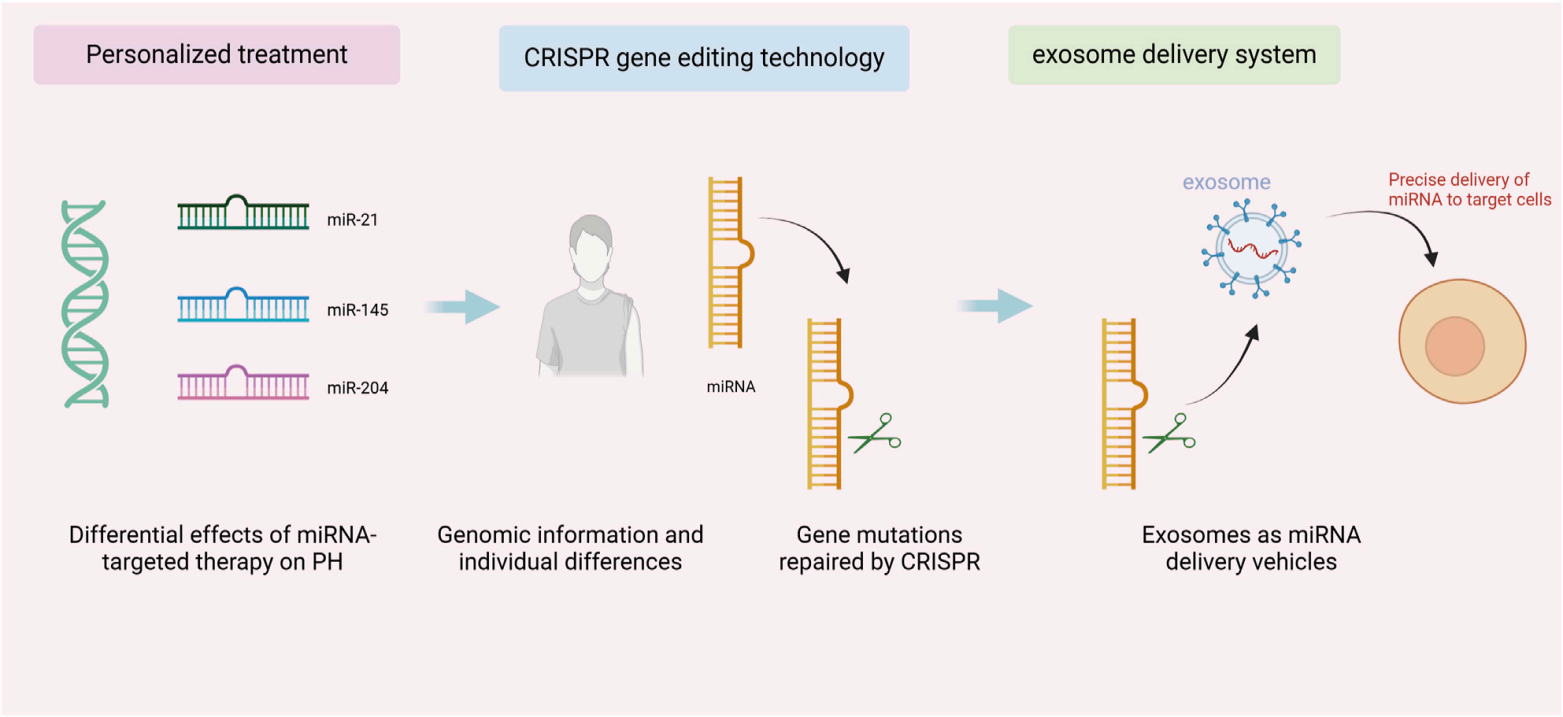
Personalized therapy and CRISPR gene editing combined with exosome delivery system for pulmonary arterial hypertension treatment.

As shown in Figure 3, personalized therapy and CRISPR gene editing combined with an exosomal delivery system, this integrated therapeutic strategy demonstrates how to advance the practical application of precision medicine in PH treatment by precisely editing genes and utilizing an exosomal delivery system. In terms of CRISPR technology, it has the ability to precisely repair miRNA expression defects, and by virtue of this advantage, it can regulate the pathogenic mechanism of PH from the root (Tong et al., 2021). Currently, researchers are significantly reducing off-target effects and improving therapeutic precision by optimizing the CRISPR system, such as enhancing Cas9 proteins and guide RNAs (Qu et al., 2023). Although exosome delivery systems offer high targeting efficiency and biocompatibility, they still face challenges in clinical applications, including high production costs and unstable quality control (Zanganeh et al., 2023). With the development of nanotechnology and exosome engineering, exosome delivery systems are expected to become more efficient and controllable in the future. Through exosome engineering, researchers can customize the carrier function of exosomes, enabling them to selectively release cargo (such as gene editing tools or miRNAs) *in vivo*. This not only enhances the targeting and precision of treatments but also allows for the maintenance of therapeutic effects through self-repair mechanisms, providing more sustained efficacy. The combination of CRISPR and exosomes will make the treatment of PH more precise and effective.

## 7 Conclusion

### 7.1 Summary of the study

This article provides a comprehensive and in-depth review of the TGF-β signaling pathway and the role of miRNAs in PH. The way they interact with each other in the pathogenesis of the disease is also explored in depth, which is expected to open up new targets for the treatment of PH. As far as the TGF-β signaling pathway is concerned, it plays a crucial role in the proliferation, migration, fibrosis, and inflammatory response of VSMCs. It acts not solely through the traditional Smad2/3 pathway but also through several nonclassical pathways (such as PI3K/Akt, MAPK, and RhoA/ROCK). Collectively, these factors drive the pathological progression of PH (Shi et al., 2022). The effects of TGF-β, once activated, are multifaceted. On one hand, it accelerates the process of vascular remodeling and fibrosis, and on the other hand, it exacerbates its own pathogenic effects even further through the interaction of the complex signaling network formed between it and miRNAs. In this regard, specific miRNAs (e.g., miR-204, miR-145, and miR-21) play important regulatory roles in the entire process. Specifically, downregulation of miR-204 results in over-activation of TGF-β signaling; high expression of miR-21 increases cell proliferation and anti-apoptotic properties, which aggravates the degree of vascular fibrosis and stiffness. In addition, upregulation of miR-145 is closely associated with vascular remodeling, fibrosis, and inflammation in PH (Eid et al., 2021; Tang et al., 2022; Deshpande et al., 2022). Therefore, restoring the expression of miR-204 to a normal state or inhibiting the overexpression of miR-21 is considered a potentially viable strategy for treating PH. In addition, CRISPR gene editing technology combined with an exosomal delivery system is an innovative therapeutic tool that can precisely edit miRNAs and their target genes (Liu B. et al., 2023; Jadamba et al., 2024). Simultaneously, exosomal delivery is highly efficient and enhances the targeting and efficacy of the therapy (Guo et al., 2023). The combination of the two opens up a new breakthrough direction for the treatment of PH and promotes the precision treatment process. Overall, the interaction between TGF-β and miRNA is appropriate, providing a new research direction for PH targeted therapy, building a solid theoretical foundation for clinical application and providing a reliable practical basis. With the continuation of these emerging technologies, the prospects for clinical application of PH therapy will become much broader.

### 7.2 Future research directions and therapeutic prospects

Future studies should focus on the in-depth exploration of the mechanism of interaction between the TGF-β signaling pathway and miRNAs, especially in the development of PH, and in particular, how miRNAs regulate TGF-β signaling. Although studies have revealed the regulatory effects of TGF-β on VSMCs through the classical Smad pathway as well as non-classical pathways, the feedback mechanism between TGF-β and miRNAs has not been fully elucidated. Therefore, future studies should focus on parsing this complex feedback mechanism and delving into the role of miRNAs in different cell types, which will help identify new biomarkers and therapeutic targets and thus promote the development of personalized therapy. In addition, with the advancement of miRNA-targeted therapeutics, research should also focus on precisely regulating miRNA expression, especially to restore protective miRNAs such as miR-204 or inhibit the overexpression of pathogenic miRNAs such as miR-21, to slow down vascular fibrosis and the inflammatory response, and to improve the pressure on the pulmonary artery and right heart function. Optimization of personalized treatment should also be the focus of future research, with genomics, transcriptomics, and single-cell analysis technologies to precisely identify differences in molecular mechanisms in patients with PH and tailor personalized treatment plans. In addition, the combined application of CRISPR gene-editing technology and exosome delivery systems will provide innovative solutions for PH treatment by precisely repairing miRNA expression defects and improving therapeutic targeting and efficacy. Finally, future research should promote the integration of clinical translation and multi-omics technologies to facilitate the clinical application of miRNA-targeted therapies to overcome the challenges of efficiency, safety, and stability of delivery systems and provide further support for personalized treatment of PH.
